# Human Antibody Isotypes, Subclasses, Allotypes and Fc Biology: Clinical Implications in Infectious Diseases, Autoimmunity, and Cancer

**DOI:** 10.3390/ph19081290

**Published:** 2026-08-15

**Authors:** Surabhi Gautam, Swarandeep Singh, Vidhi Thakkar, Devyani Joshi, Sanjeev Kumar

**Affiliations:** 1 Department of Anatomy, Faculty of Medicine and Health Sciences, SGT University, Gurugram 122505, Haryana, India; 2 Department of Biochemistry, All India Institute of Medical Sciences, New Delhi 110029, Delhi, India; swarandeep@aiims.edu (S.S.); kumarsanjeevdr1@gmail.com (S.K.); 3 Department of Pharmaceutical Sciences, School of Pharmacy, Massachusetts College of Pharmacy and Health Sciences, Boston, MA 02115, USA; vthak3@stu.mcphs.edu

**Keywords:** Immunoglobulin isotypes, IgG subclasses, allotypes, Fc receptors, FcRn, glycosylation, monoclonal antibodies, pharmacokinetics, pharmacodynamics, drug delivery, biopharmaceutics, infectious diseases, autoimmunity, cancer

## Abstract

Therapeutic antibodies have transformed the management of infectious diseases, autoimmune disorders, and cancer, yet most approved agents continue to rely on a limited range of IgG-based Fc scaffolds. Consequently, the broader therapeutic potential of antibody isotypes, subclasses, allotypes, and host Fc receptor (FcR) genetics remains incompletely integrated into antibody design and clinical development. Emerging evidence indicates that these determinants profoundly influence effector function, pharmacokinetics, tissue distribution, immunogenicity, and therapeutic efficacy. This review provides a comprehensive, translational perspective on how antibody isotype selection, IgG subclass biology, allotypic variation, Fcγ receptor (FcγR) and neonatal Fc receptor (FcRn) genetics, and Fc engineering collectively shape therapeutic outcomes. Distinct from previous reviews that examine these factors individually, we integrate their roles across infectious diseases, autoimmunity, and cancer to identify common principles governing Fc-dependent immunity and therapeutic response. We further describe the clinical implications of Fc-mediated mechanisms, Fc glycoengineering, FcRn-targeted therapies, systems serology, and emerging non-IgG antibody platforms, highlighting both established applications and unresolved challenges. By unifying structural immunology, Fc pharmacology, host genetics, and disease biology, this review advances the concept of precision Fc pharmacology, in which antibody scaffold selection and Fc optimization are tailored to specific disease contexts and patient immune profiles. This framework provides a roadmap for the rational development of next-generation antibody therapeutics with improved efficacy, safety, and clinical precision.

## 1. Introduction

Monoclonal antibodies (mAbs) and Fc fusion proteins have become a cornerstone of modern therapeutics, accounting for a substantial proportion of recent drug approvals across oncology, autoimmune and inflammatory diseases, transplantation, and infectious diseases [1]. Their clinical success derives from the combination of antigen specificity mediated by the variable region and the diverse biological functions conferred by the Fc domain, which regulates interactions with Fc receptors (FcRs), complement proteins, and the neonatal FcR (FcRn). These interactions determine key therapeutic properties, including antibody-dependent cellular cytotoxicity (ADCC), antibody-dependent cellular phagocytosis (ADCP), complement-dependent cytotoxicity (CDC), serum half-life, tissue distribution, and immunomodulatory activity [2,3]. Despite major advances in antibody engineering, most approved therapeutics continue to rely on a relatively narrow range of Fc scaffolds, predominantly human IgG1, with more limited use of alternative subclasses such as IgG2 for agonistic receptor targeting and IgG4 for checkpoint inhibitors and anti-cytokine therapies [1,4].

Human antibodies comprise five major isotypes (IgM, IgD, IgG, IgA, and IgE), each possessing distinct structural and functional properties that influence immune responses and therapeutic potential [2,3]. Within the IgG isotype, the four subclasses differ in hinge architecture, FcR engagement, complement activation, and pharmacokinetic behavior, resulting in distinct capacities to mediate cytotoxicity, phagocytosis, and inflammatory signaling [3,4]. Beyond isotype and subclass diversity, naturally occurring allotypic variation within IgG constant regions and polymorphisms in Fcγ receptor (FcγR) and FcRn genes introduce an additional layer of biological complexity that can influence antibody binding, effector function, pharmacokinetics, vaccine responsiveness, and clinical outcomes [3,4,5].

**Figure 1:**
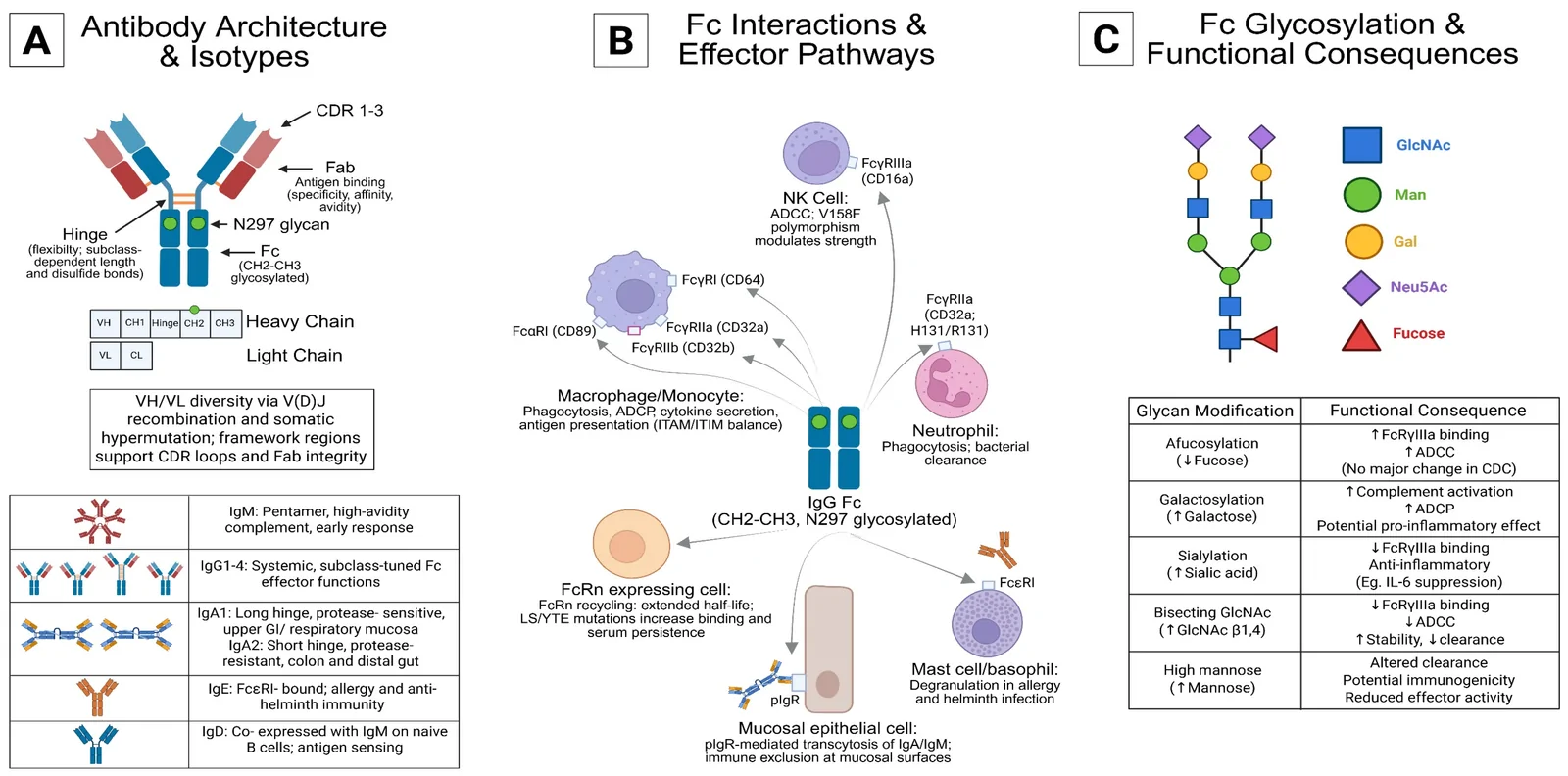
Integrated overview of antibody structure, Fc interactions, and glycosylation-dependent effector functions. (**A**) Antibody architecture and isotypes. A generic IgG molecule is shown with paired variable heavy (VH) and light (VL) domains forming the Fab region that mediates antigen binding (specificity, affinity, and avidity), supported by conserved framework regions and hinge flexibility. The Fc region (CH2-CH3) bears a core N297 glycan that is critical for FcR and complement engagement. Below, the five major human isotypes (IgM, IgG1–4, IgA1/2, IgE, IgD) are summarized with their dominant structural features and immunological niches. (**B**) Fc interactions and effector pathways. The central IgG Fc fragment (CH2–CH3, N297 glycosylated) engages a network of FcRs on innate and adaptive immune cells, including FcγRI (CD64), FcγRIIa/c (CD32a/c), FcγRIIb (CD32b), FcγRIIIa (CD16a), FcαRI (CD89), FcεRI, the FcRn, and the polymeric Ig receptor (pIgR). (**C**) Fc glycosylation and functional consequences. Schematic N-glycan structures illustrate the principal Fc glycan components (GlcNAc, mannose, galactose, sialic acid, fucose) and a summary table links common Fc glycan modifications to functional outcomes. Created in BioRender. Joshi, D. (2026) https://BioRender.com/71x0ct2.

Most existing reviews examine antibody isotypes, subclasses, FcR biology, Fc engineering, or disease applications individually and do not provide an integrated framework linking these complementary sources of antibody diversity to therapeutic design and clinical decision-making across major disease settings [2,4,6,7]. Furthermore, emerging insights from systems serology, including antibody isotype and subclass profiling, Fc glycosylation patterns, and Fc-mediated functional signatures, have yet to be fully incorporated into the rational development of therapeutic and prophylactic antibodies [8]. This review addresses this gap by providing a comprehensive, disease-oriented synthesis of human antibody isotypes, IgG subclasses, allotypes, FcR biology, and Fc-mediated mechanisms across infectious diseases, autoimmunity, and cancer. Importantly, we integrate advances in Fc engineering, pharmacogenomics, FcRn biology, and systems immunology to examine how antibody scaffold selection and Fc optimization can be tailored to specific disease contexts. By integrating structural immunology, Fc receptor biology, host immunogenetics, clinical pharmacology, and disease-specific immune profiling, we define precision Fc pharmacology as a rational framework for selecting antibody isotype/subclass, Fc glycosylation, Fc engineering strategy, and FcR engagement according to the immune mechanisms driving a given disease. Rather than optimizing any single Fc property in isolation, this framework considers these variables collectively to maximize therapeutic efficacy while minimizing toxicity. As illustrated in Figure 1, disease biology first defines the desired effector mechanism, which subsequently guides isotype/subclass selection, Fc glycosylation, Fc engineering, and receptor engagement, while host FcγR/FcRn polymorphisms and allotypes act as modifying determinants of therapeutic response.

## 2. Fundamental Structure and Diversity of Human Antibody Isotypes, Subclasses, and Allotypes

### 2.1. Immunoglobulin Architecture and Antibody Isotypes

The therapeutic activity of antibodies is determined by the coordinated functions of their antigen-binding and Fc domains. The structural and functional relationships are summarized schematically in Figure 1A, which highlights the division between Fab-mediated antigen recognition and Fc-dependent effector functions across human isotypes. Human immunoglobulins are heterodimeric glycoproteins composed of two identical heavy chains and two identical light chains arranged into a Y-shaped structure, in which paired heavy- and light-chain complementarity-determining regions (CDRs) form the antigen-binding site. At the same time, the constant domains govern effector function and pharmacological behavior [2]. The heavy-chain constant region defines antibody isotype and thereby regulates interactions with FcRs, complement components, and FcRn, which collectively influence immune activation, tissue distribution, and serum persistence. Through class-switch recombination, B cells can preserve antigen specificity while coupling the same variable region to distinct Fc architectures, allowing identical antigen recognition to generate markedly different biological outcomes [2]. This principle underlies many contemporary antibody-engineering strategies and highlights the therapeutic importance of Fc diversity [2].

### 2.2. Functional Diversity of Human Antibody Isotypes

Human antibodies are classified into five major isotypes: IgM, IgD, IgG, IgA, and IgE, each adapted to distinct immunological niches and effector functions (Table 1). IgM is typically produced as a pentameric or hexameric molecule containing a joining (J) chain and is particularly effective at activating the classical complement pathway during primary immune responses [2]. IgD is primarily expressed on naïve B cells and remains the least understood isotype with respect to soluble effector functions [2]. IgG is the predominant serum immunoglobulin and mediates diverse functions, including neutralization, opsonization, FcγR engagement, and complement activation, making it the principal scaffold for therapeutic antibodies [2,3]. IgA exists as serum monomers and secretory dimers, providing specialized protection at mucosal surfaces through immune exclusion and pathogen neutralization [2]. In contrast, IgE binds with high affinity to Fc epsilon receptor-1 (FcεRI) on mast cells and basophils, where antigen-induced crosslinking triggers degranulation and inflammatory responses that contribute to allergic disease and anti-helminth immunity [2,3]. Although IgG dominates the current therapeutic landscape, growing interest in IgA-, IgM-, and IgE-based therapeutics reflects recognition that different isotypes possess unique functional properties that may be advantageous in specific disease settings.

### 2.3. Antibody Subclasses as Determinants of Therapeutic Function

#### 2.3.1. IgG Subclasses

Among human antibody classes, IgG exhibits the greatest therapeutic relevance and functional diversity. The four human IgG subclasses (IgG1, IgG2, IgG3, and IgG4) share high sequence homology yet differ in hinge structure, FcγR engagement, complement activation, and pharmacokinetic behavior (Table 1) [3]. These differences translate directly into distinct biological activities and have important implications for antibody design. IgG1 and IgG3 generally display the strongest interactions with activating FcγRs and efficiently mediate ADCC and ADCP, while also supporting complement activation through C1q binding [3,4]. The extended hinge region of IgG3 enhances Fc accessibility and effector activity but is associated with increased proteolytic susceptibility and a shorter serum half-life [3,4].

In contrast, IgG2 and IgG4 exhibit weaker FcγR engagement and reduced inflammatory potential, making them attractive platforms when potent cytotoxic activity is undesirable [3,4]. IgG4 possesses the additional ability to undergo Fab-arm exchange, generating functionally monovalent antibodies with diminished crosslinking capacity. The therapeutic significance of these subclass differences has been demonstrated in studies comparing antibodies with identical antigen-binding regions but different Fc subclasses, in which Fc-mediated functions varied substantially despite unchanged antigen specificity [4]. Moreover, structural analyses have identified residues within the lower hinge and CH2 domains that selectively regulate FcγR versus complement interactions, providing opportunities to engineer antibodies that enhance, attenuate, or decouple specific effector mechanisms [3,9]. Collectively, these observations establish that subclass selection is a critical, often underappreciated design variable in therapeutic antibody development.

#### 2.3.2. IgA Subclasses

IgA is the dominant antibody at mucosal surfaces and plays a critical role in barrier immunity, pathogen neutralization, and immune homeostasis. Human IgA comprises two subclasses, IgA1 and IgA2, which differ in hinge-region structure, glycosylation, tissue distribution, and functional specialization (Table 1) [10,11]. IgA1 accounts for approximately 80–90% of serum IgA and contains a long, heavily O-glycosylated hinge region that provides enhanced flexibility for antigen binding. However, this extended hinge is susceptible to cleavage by bacterial IgA1-specific proteases. In contrast, IgA2 possesses a shorter, protease-resistant hinge region and is enriched in the distal gastrointestinal tract, where microbial density and proteolytic activity are high [10,11]. Both subclasses exist as monomers in circulation and as J-chain-mediated dimers in mucosal secretions. Dimeric IgA is transported across epithelial cells by the polymeric immunoglobulin receptor (pIgR), generating secretory IgA, which provides durable protection at mucosal surfaces [12]. IgA mediates immune exclusion by preventing pathogen adherence and can neutralize microorganisms during epithelial transcytosis. Through interaction with FcαRI (CD89) on neutrophils, monocytes, and macrophages, IgA also promotes phagocytosis, degranulation, and cytokine release [13]. The clinical relevance of IgA subclasses is highlighted by the association of aberrantly glycosylated IgA1 with IgA nephropathy, whereas IgA2 plays an important role in intestinal mucosal defense [14,15]. Growing interest in IgA-based therapeutics reflects its unique ability to recruit myeloid effector cells and protect mucosal barriers, making it an attractive platform for future vaccines and antibody therapies.

### 2.4. IgG Allotypes and Functional Heterogeneity

In addition to isotype and subclass diversity, inherited polymorphisms within immunoglobulin constant regions contribute to interindividual variation in antibody function. Human IgG heavy chains express genetically encoded immunoglobulin γ marker (GM) allotypes, while analogous KM (κ marker) allotypes occur within light-chain constant regions [3]. These polymorphisms differ in frequency among populations and can influence Fc structure, receptor interactions, and complement engagement [3]. Early serological and genetic studies associated specific GM allotypes with susceptibility to infectious diseases and differences in vaccine responsiveness. However, many findings require validation in contemporary cohorts using standardized methodologies and appropriate control for population stratification [3]. More recent mechanistic studies have begun to define the functional consequences of these variants. Using recombinant IgG1, IgG2, IgG3, and IgG4 antibodies containing distinct GM allotypes but identical antigen-binding regions, investigators demonstrated modest yet measurable differences in FcγR binding. However, these effects were often less pronounced in cell-based effector assays [3]. Similarly, complement-focused analyses of 27 IgG allotypes directed against trinitrophenyl revealed subclass-dependent effects on complement binding, deposition, and complement-dependent cytotoxicity, with some IgG2 allotypes displaying greater functional variability than corresponding IgG1, IgG3, or IgG4 variants [16]. Current evidence therefore suggests that allotypic variation functions primarily as a quantitative modifier of Fc-dependent biology rather than a dominant determinant of therapeutic activity [3,5,16]. Nevertheless, the growing integration of pharmacogenomics, Fc engineering, and precision medicine raises the possibility that allotype-specific effects may become increasingly relevant for therapeutic optimization, biomarker discovery, and individualized antibody treatment strategies.

## 3. Fc Receptors, FcRn, and Core Effector Mechanisms

### 3.1. FcγRs as Determinants of Antibody Effector Function

The biological activity of therapeutic antibodies is critically influenced by their interactions with FcγRs, which translate antibody recognition of target antigens into cellular immune responses [3,7,17]. Human FcγRs comprise the high-affinity receptor FcγRI (CD64) and the low-to-intermediate-affinity receptors FcγRIIa (CD32a), FcγRIIb (CD32b), FcγRIIc (CD32c), FcγRIIIa (CD16a), and FcγRIIIb (CD16b), each exhibiting distinct cellular expression patterns, signaling properties, and subclass-binding preferences (Table 2) [3,4]. Activating FcγRs signal through immunoreceptor tyrosine-based activation motifs (ITAMs), whereas FcγRIIb contains an inhibitory immunoreceptor tyrosine-based inhibitory motif (ITIM), creating a regulatory balance that shapes the magnitude and quality of Fc-mediated immune responses [3,4]. Biophysical and cellular studies have demonstrated that IgG subclasses differ substantially in their capacity to engage FcγRs, with IgG3 generally exhibiting the strongest affinity for activating receptors, followed by IgG1, whereas IgG2 and IgG4 display weaker interactions and reduced effector potential [4,6]. These differences have direct therapeutic implications. IgG1 remains the preferred scaffold for antibodies designed to eliminate tumor cells or infected cells through ADCC and ADCP. At the same time, IgG2 and IgG4 are frequently selected for cytokine-neutralizing or receptor-blocking therapies where excessive effector activity may be undesirable [3,4]. In parallel, polymorphisms within FcγRIIa and FcγRIIIa can alter receptor affinity and influence clinical responses to antibody therapy, highlighting the growing importance of host immunogenetics as a modifier of therapeutic efficacy [3,4]. Collectively, FcγR biology illustrates how identical antigen specificity can produce markedly different biological outcomes depending on Fc architecture and host receptor genotype, providing a strong rationale for Fc-guided therapeutic design. The key FcRs, their cellular distribution, and the resulting effector pathways are depicted in Figure 1B.

### 3.2. FcRn and the Pharmacology of Therapeutic Antibodies

Beyond effector function, therapeutic success depends on maintaining adequate systemic exposure. The FcRn is the principal regulator of IgG homeostasis and represents one of the most important determinants of antibody pharmacokinetics [3]. FcRn binds IgG within acidic endosomal compartments and releases it at physiological pH, rescuing antibodies from lysosomal degradation and recycling them back into the circulation [2,3]. This process underlies the prolonged serum half-life of IgG and contributes to maternal–fetal antibody transfer, mucosal transport, and immune-complex handling across multiple tissues [2,3]. FcRn is also expressed in myeloid cells, where it can influence antigen presentation and immune complex handling, and interspecies differences in FcRn expression and binding properties have important implications for translating Fc-engineered antibodies from animal models to humans [3,18]. The translational significance of FcRn has become increasingly evident through both Fc engineering and therapeutic FcRn blockade. Mutations such as YTE and LS enhance FcRn engagement at acidic pH and have been incorporated into long-acting antibody platforms to extend serum persistence and reduce dosing frequency [7]. Conversely, antagonistic antibodies and Fc fragments that block FcRn accelerate IgG catabolism and are emerging as effective therapies for autoantibody-mediated diseases [17,18]. Importantly, species-specific differences in FcRn expression and binding characteristics remain a major challenge in the preclinical evaluation of Fc-engineered antibodies and must be considered when translating findings from animal models to humans [3,18]. These advances have transformed FcRn from a passive determinant of antibody persistence into a tractable therapeutic target and a central design parameter in modern antibody development.

### 3.3. Fc-Mediated Effector Mechanisms and Therapeutic Context

Following antigen recognition, antibody Fc domains can recruit immune cells and complement proteins to eliminate pathogens, malignant cells, or pathogenic host targets [3]. Among these mechanisms, ADCC is primarily mediated by FcγRIIIa-expressing natural killer cells and, to a lesser extent, macrophages, resulting in perforin- and granzyme-dependent killing of antibody-opsonized targets [3,4]. ADCP is mediated by macrophages, neutrophils, and other FcγR-expressing phagocytes that engulf and degrade antibody-coated cells or microorganisms [3]. CDC is initiated when C1q binds clustered Fc domains on antigen-bearing surfaces, triggering activation of the classical complement cascade and subsequent formation of the membrane attack complex [3,16]. The efficiency of each pathway is strongly influenced by antibody subclass, Fc geometry, glycosylation state, antigen density, and local tissue environment [3,4]. The relative contribution of these mechanisms varies considerably across therapeutic indications. In oncology, IgG1 antibodies that efficiently engage FcγRIIIa on natural killer cells and macrophages are often favored because they promote ADCC and ADCP [4,7].

In contrast, antibodies intended to neutralize soluble cytokines or block immune receptors in chronic inflammatory diseases often employ IgG2, IgG4, or Fc-silenced formats to minimize unintended depletion of target-expressing cells and improve safety [4,7]. Emerging evidence further indicates that Fc-mediated activity is shaped not only by antibody subclass and receptor interactions but also by Fc glycosylation and system-level immune context. Systems serology approaches that simultaneously evaluate isotype, subclass, Fc glycosylation, and effector function have identified multidimensional antibody signatures associated with protection, vaccine responsiveness, and disease severity [8]. These findings support a transition from single-parameter antibody optimization toward integrated Fc design strategies that account for both molecular architecture and host immune biology. Figure 1C summarizes major Fc glycan structures and highlights how specific glycan modifications modulate FcγRIIIa binding, ADCC, complement activation, and clearance profiles.

## 4. Therapeutic Antibody Landscape: Biological Diversity and Clinical Convergence

The rapid expansion of antibody therapeutics has transformed multiple areas of medicine; however, analysis of approved and clinically developed antibodies reveals a striking disconnect between the extensive natural diversity of human immunoglobulins and the comparatively narrow range of Fc architectures used in clinical practice. A systematic survey of 819 antibody-related therapeutics and Fc fusion proteins assigned World Health Organization (WHO) international nonproprietary names identified 804 molecules developed for therapeutic use, including 719 conventional mAbs, 55 Fc-fusion proteins, and 20 Fc-containing fusion constructs with alternative targeting modules [1]. Despite the availability of multiple isotypes, subclasses, and allotypic variants, therapeutic development remains heavily concentrated within a small subset of IgG scaffolds. Among 606 therapeutic IgG molecules with fully characterized sequences, 409 were derived from human IgG1, 131 from IgG4, and 48 from IgG2, whereas only a single therapeutic IgG3 molecule was identified [1]. Similarly, allotypic diversity was highly restricted, with IGHG1*01, IGHG1*03, and closely related variants accounting for approximately 96% of human IgG1 therapeutics, while IGHG2*01 and IGHG4*01 predominated among IgG2 and IgG4 products [1]. Kappa light-chain usage showed comparable convergence, with Km3 representing the overwhelming majority of human kappa sequences [1]. These findings suggest that therapeutic antibody development has largely favored a limited set of well-characterized constant-region templates despite substantial naturally occurring immunoglobulin diversity.

A recent review identified 91 clinically used mAbs, the majority of which were IgG1 molecules targeting tumor-associated antigens or immune checkpoints, with limited representation of IgG2, IgG4, and non-IgG formats [19]. Likewise, broader analyses of approved antibodies have emphasized the continued dominance of IgG1- and IgG4-based platforms with relatively conserved Fc glycosylation profiles despite increasing interest in alternative Fc architectures [20]. Collectively, these observations indicate that clinical antibody development has historically prioritized manufacturability, regulatory familiarity, and predictable pharmacology over exploration of the full spectrum of antibody isotype and subclass biology [1,19,20].

At the same time, advances in protein engineering have substantially expanded the range of Fc modifications available for therapeutic optimization. Analysis of the same dataset identified 57 molecular formats and approximately 90 distinct constant-region variants, including 47 designs intended to reduce effector activity, 14 to enhance Fc-mediated functions, eight to modify FcRn interactions, and 13 to facilitate heavy-chain heterodimerization [1]. Among 756 therapeutics containing intact Fc domains, 339 (approximately 45%) incorporated either naturally low-effector IgG2 or IgG4 backbones or Fc-engineered variants designed to reduce FcγR and complement engagement, demonstrating that effector silencing has become nearly as common as effector enhancement in modern antibody development [1]. These engineering strategies generally fall into three major categories: enhancement of FcγR or C1q interactions to increase ADCC and CDC, attenuation of Fc-mediated effector functions to improve safety or preserve target cells, and modulation of FcRn binding to alter pharmacokinetic properties [7]. Notably, only a small fraction of the many Fc variants reported in experimental studies have progressed into approved therapeutics. Examples include afucosylated IgG1 molecules with enhanced FcγRIIIa binding, Fc-engineered antibodies containing substitutions such as S239D and I332E to increase effector potency, and half-life extension variants incorporating YTE or LS mutations that enhance FcRn-mediated recycling [7].

The therapeutic importance of Fc optimization is particularly evident when comparing distinct clinical indications. In oncology, the clinical activity of anti-CD20, anti-HER2, and anti-CD38 antibodies has reinforced the value of FcγR engagement, complement activation, and Fc-enhanced antibody–drug conjugates (ADCs) for maximizing tumor cell elimination [20,21,22]. In contrast, therapies for autoimmune and inflammatory diseases frequently employ IgG2, IgG4, or Fc-silenced IgG1 backbones to minimize unwanted depletion of target-expressing cells and limit off-target inflammation. At the same time, FcRn antagonists have emerged as a complementary strategy for reducing pathogenic IgG burden [7]. In infectious diseases, systems serology studies have demonstrated that protective immunity is often associated with coordinated patterns of antibody subclass usage, Fc glycosylation, phagocytic activity, complement deposition, and mucosal effector functions. Yet, most licensed anti-infective mAbs continue to rely on conventional IgG1 Fc frameworks [8,21,22]. Taken together, the current therapeutic landscape highlights a central paradox in antibody drug development: while technological capabilities now permit extensive manipulation of antibody isotypes, subclasses, allotypes, glycosylation profiles, and FcR interactions, clinical products remain concentrated within a remarkably limited set of Fc scaffolds. This gap between biological diversity and therapeutic implementation represents a major opportunity for innovation.

## 5. Isotypes and IgG Subclasses as Deliberate Design Variables

Historically, antibody isotypes and subclasses were often selected based on platform familiarity and manufacturability rather than explicit therapeutic rationale. Increasing mechanistic and clinical evidence now supports a more deliberate approach in which Fc architecture is considered a critical design variable that influences efficacy, safety, tissue distribution, and immune engagement [3,4]. Although direct clinical comparisons between subclasses remain uncommon, accumulated data from therapeutic antibodies and engineered Fc variants have established several broad principles that can guide indication-specific antibody design. For therapies requiring efficient elimination of target cells, IgG1 remains the preferred backbone because of its capacity to engage activating FcγRs and support ADCC, ADCP, and, in some settings, complement activation [4]. Accordingly, antibodies targeting CD20, HER2, and CD38 commonly employ IgG1 Fc regions to maximize immune-mediated tumor cell killing [20].

In contrast, IgG2 is frequently favored for agonistic antibodies that rely on receptor clustering rather than cellular depletion, whereas IgG4 is widely used for receptor-blocking and immune-modulating therapies because of its comparatively reduced effector activity [4]. Clinical examples include the immune checkpoint inhibitors pembrolizumab and nivolumab, which utilize engineered IgG4 backbones with hinge-stabilizing modifications to minimize FcγR-mediated depletion of activated T cells while preserving target blockade [20]. These examples illustrate how subclass selection can be used to align Fc function with therapeutic mechanisms.

A similar design logic is evident in autoimmune and inflammatory diseases, where the primary objective is often the neutralization of cytokines or the interruption of pathogenic signaling pathways rather than the elimination of target-expressing cells. Consequently, many anti-TNF, anti-IL-6 receptor, and anti-integrin antibodies employ IgG4 scaffolds, IgG2 backbones, or Fc-engineered IgG1 variants containing silencing mutations, such as LALA (L234A/L235A), while stabilizing substitutions, such as S228P, are commonly incorporated into IgG4 molecules [7]. These approaches reduce FcγR engagement and complement activation, thereby limiting unintended inflammatory consequences while preserving therapeutic activity [7]. Collectively, these observations support a general pharmacological principle whereby effector-competent Fc regions are favored for depleting therapies. In contrast, effector-silent or attenuated Fc domains are preferred for blockade-based interventions.

Beyond conventional IgG therapeutics, alternative antibody isotypes offer opportunities to access biological functions that are not readily achieved using existing IgG platforms. Secretory IgA, through engagement of FcαRI and transport across mucosal surfaces, plays a central role in immune exclusion and pathogen neutralization at epithelial barriers [2]. Pentameric IgM provides exceptionally high avidity and potent complement activation, properties that may be advantageous in settings requiring rapid pathogen clearance [2]. These features have stimulated growing interest in IgA- and IgM-based therapeutics for mucosal infections and selected malignancies [19]. Similarly, IgE-based strategies seek to exploit tissue-resident mast cells, eosinophils, and other FcεRI-expressing effector cells to enhance antitumor immunity. However, clinical experience remains limited to early-stage studies and small clinical trials [19,23]. Despite these unique biological advantages, broader clinical translation of IgA-, IgM-, and IgE-based therapeutics is constrained by several important challenges, including shorter serum half-lives due to limited FcRn-mediated recycling (for IgA and IgM), structural complexity and product heterogeneity associated with multimerization and glycosylation, difficulties in scalable manufacturing and quality control, and safety concerns such as FcεRI-mediated hypersensitivity with IgE and FcαRI-driven inflammatory activation with IgA, all of which have limited clinical development beyond early-phase studies [19,23].

The emergence of systems serology further reinforces the need to view antibody architecture through a disease-specific lens. Rather than focusing solely on antigen binding or neutralization, systems serology integrates measurements of antibody isotype, subclass, Fc glycosylation, FcR engagement, complement deposition, phagocytic activity, and natural killer cell activation to define multidimensional correlates of protection and pathology [8]. Studies in HIV, influenza, and other infectious diseases have demonstrated that protective immunity frequently depends on coordinated Fc-mediated functions beyond neutralizing activity, with favorable outcomes associated with antibody profiles that promote efficient opsonophagocytosis, balanced FcγR engagement, and beneficial glycosylation patterns [8]. These findings suggest that optimal antibody formats may differ substantially across infectious diseases, cancer, and autoimmunity and support incorporating systems-level immune profiling into early-stage therapeutic development. Taken together, the limited clinical exploitation of this diversity, combined with the scarcity of prospective subclass-comparison studies, highlights a major opportunity for more systematic, indication-driven antibody design. Such an approach forms a central theme of this review and provides a foundation for the precision Fc pharmacology framework developed in subsequent sections.

## 6. Allotypes and FcγR as Determinants of Clinical Heterogeneity

The clinical performance of therapeutic antibodies is influenced not only by antibody structure and Fc engineering but also by inherited variation within the host immune system. Genetic polymorphisms in immunoglobulin constant regions and FcγR affect interindividual differences in Fc-mediated biology, potentially influencing therapeutic efficacy, pharmacokinetics, and safety. Despite increasing recognition of these effects, host immunogenetic variation remains underutilized in antibody development and clinical decision-making, representing a major opportunity to advance precision Fc pharmacology.

### 6.1. Immunoglobulin Allotypes and Functional Diversity

Among the earliest recognized sources of antibody genetic diversity are immunoglobulin allotypes, inherited polymorphisms encoded within constant-region genes. Classical studies established that immunoglobulin GMs (γ markers) correspond to amino-acid differences within IgG subclasses and exhibit substantial population-specific variation [3]. More recent analyses suggest that these polymorphisms may contribute to differences in susceptibility to infectious diseases, vaccine responsiveness, and humoral immunity across populations [24,25]. These genetic variants can influence antibody responses against microbial, autoantigenic, and tumor-associated antigens, although many reported associations remain based on relatively small cohorts and require validation in larger populations [24,25]. Importantly, the potential significance of allotypic variation extends beyond antibody quantity to functional quality. A case–control study investigating immune responses to a conserved microbial polysaccharide in patients with chronic pulmonary disease demonstrated that specific GM and KM genotypes, particularly when combined with a common FcγRIIa variant, were associated with differences in IgG subclass distribution and opsonophagocytic activity [24]. These findings suggest that inherited variation may shape both antibody composition and Fc-mediated function. Functional studies using recombinant IgG allotypic variants provide mechanistic support for this concept. Experimental analyses have shown that certain allotypic substitutions can alter FcγR binding and complement activation, although the effects are generally modest and highly context-dependent [5,16]. Collectively, these observations suggest that immunoglobulin allotypes may contribute to clinically relevant variability in antibody function. Still, current evidence remains insufficient to support routine incorporation of allotype information into therapeutic design or patient management.

### 6.2. FcγR Polymorphisms, Fc Pharmacogenomics and Precision Antibody Therapy

Among known host genetic determinants, polymorphisms in FcγR genes provide some of the strongest evidence for a link between inherited variation and therapeutic antibody outcomes. FcγRs are central mediators of antibody-dependent effector functions, and genetic variants affecting receptor affinity can alter interactions with therapeutic antibodies and endogenous immunoglobulins. Seminal clinical studies of rituximab demonstrated that patients carrying the high-affinity FcγRIIIa V158 allele achieved superior clinical responses compared with individuals homozygous for the lower-affinity F158 variant, supporting a direct role for FcγR-mediated effector mechanisms in therapeutic efficacy [3,26]. Subsequent investigations identified additional variants within FcγRIIa and FcγRIIIb that influence subclass-specific binding and functional responses [4,20,22]. Across oncology and autoimmune diseases, these polymorphisms have been associated with variation in ADCC and ADCP, as well as clinical outcomes, although reported effect sizes vary across studies and disease settings [4,20,22]. The translational implications of these findings are substantial. Host FcγR genotype may partially explain why patients receiving identical therapeutic antibodies can experience markedly different clinical responses. Nevertheless, routine FcγR genotyping has not been adopted in clinical practice because available evidence remains inconsistent, and prospective validation studies are limited. Instead, many contemporary Fc-engineering strategies attempt to overcome host genetic variability directly. Approaches such as afucosylation and Fc variants with enhanced FcγRIIIa binding are designed to strengthen receptor engagement across multiple genetic backgrounds, thereby reducing dependence on favorable FcγR genotypes [7,22]. This strategy reflects a broader trend in antibody engineering: rather than tailoring therapy to host genetics, current development efforts often aim to create Fc architectures that perform robustly despite underlying genetic heterogeneity. Taken together, evidence from immunoglobulin allotypes and FcγR polymorphisms suggests that host genetics contributes meaningfully to variability in antibody-mediated immunity and therapeutic responses [3,4,24]. For most specific combinations of antibody allotype, FcR genotype, disease indication, and therapeutic antibody, available data are insufficient to justify formal genotype-based prescribing recommendations [18,25]. This gap highlights a major opportunity for future research.

## 7. Disease-Specific Fc Biology in Infectious Diseases

**Figure 2:**
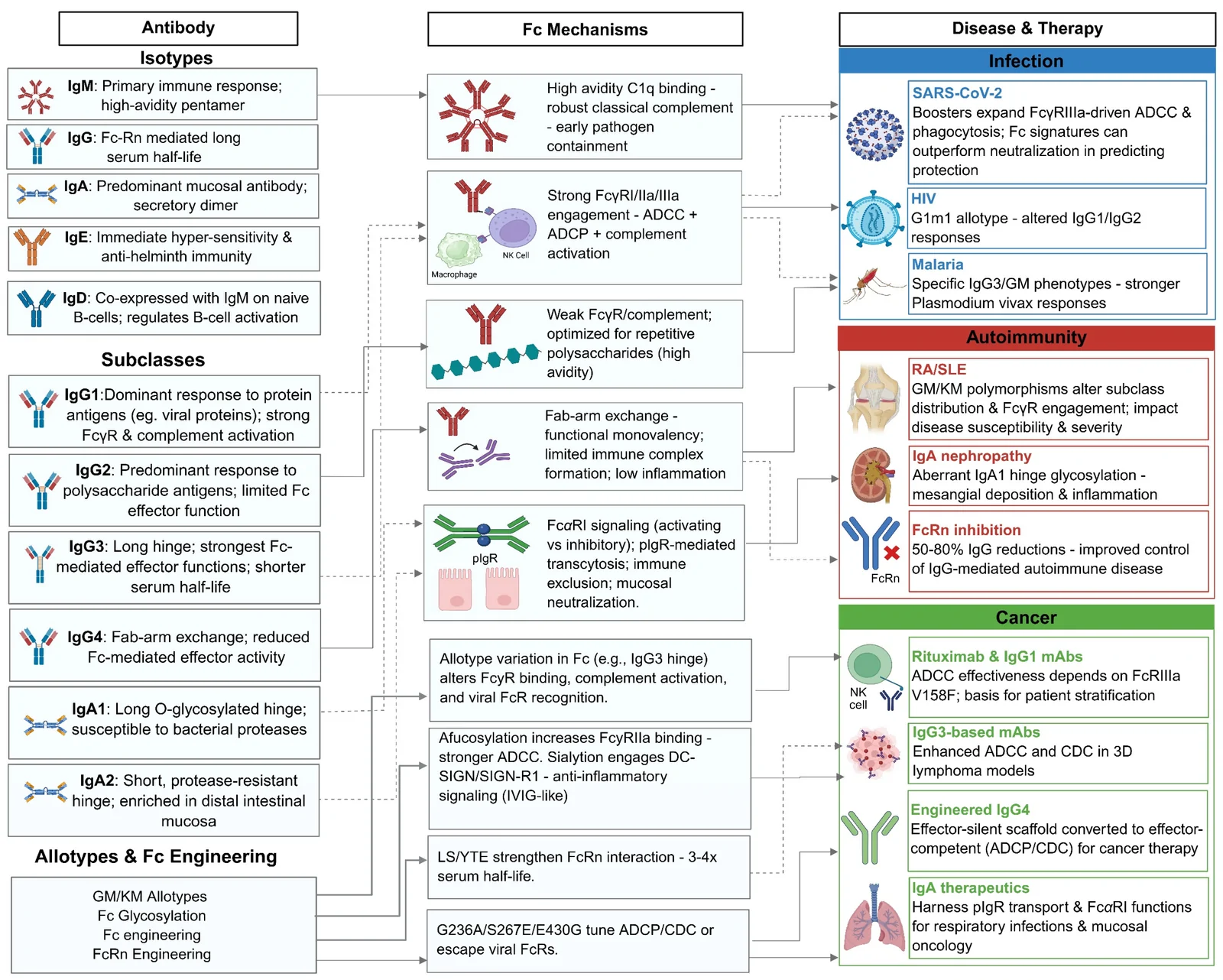
Linking antibody isotypes, subclasses, allotypes, and Fc engineering to Fc mechanisms and disease-specific therapeutic applications. The left column summarizes key biological properties of human antibody isotypes, subclasses, and Fc variants. The bottom left panel highlights GM/KM allotypes, Fc glycoforms, FcRn-modifying mutations, and effector-tuning substitutions as key Fc-engineering levers that modulate Fcγ receptor binding, complement activation, half-life, and escape from viral FcRs. The middle column depicts the corresponding Fc mechanisms. The right column links these Fc mechanisms to representative disease settings and therapeutic examples in infection, autoimmunity, and cancer. Created in BioRender. Joshi, D. (2026) https://BioRender.com/71x0ct2.

For the following sections, Figure 2 shows antibody isotypes, IgG subclasses, allotypes, and Fc engineering with their core Fc mechanisms and illustrates how these combinations translate into indication-specific requirements across infectious diseases, autoimmunity, and cancer.

### 7.1. Protective Fc-Mediated Functions Beyond Neutralization

A growing body of evidence demonstrates that protection against infectious diseases is not determined solely by antibody neutralization but also by Fc-dependent mechanisms that influence pathogen clearance, immune regulation, and tissue protection [27]. Influenza provides one of the clearest examples of this concept. Experimental challenge studies, passive-transfer models, and intravenous immunoglobulin investigations have shown that antibodies with potent Fc-mediated functions can reduce viral burden and disease severity even when neutralizing activity is modest [28]. Protective responses are frequently enriched for IgG1 and IgG3 antibodies that engage FcγRIIIa-dependent effector pathways, suggesting that Fc optimization may be essential for next-generation universal influenza vaccines and broadly protective therapeutic antibodies [28,29,30]. A similar paradigm has emerged for respiratory syncytial virus (RSV), where neutralizing antibody (NAb) titers correlate only partially with protection [31]. The COVID-19 pandemic further accelerated recognition of Fc biology as a determinant of antiviral immunity [32]. Studies of SARS-CoV-2 demonstrated that non-NAbs targeting the spike N-terminal domain can confer protection in animal models through Fc-dependent mechanisms, with Fc-silencing mutations abolishing efficacy and Fc-enhancing modifications augmenting protection [33]. Clinical investigations similarly revealed that Fc-mediated functional profiles, rather than neutralization alone, contribute to disease modulation across age groups and may influence both natural and vaccine-induced immunity [33,34].

Beyond viral infections, tuberculosis (TB) has emerged as an important example of Fc-dependent immune regulation. Although antibodies were historically considered secondary contributors to TB control, recent studies have identified distinct isotype, subclass, and glycosylation signatures associated with latent infection, active disease, and differential disease progression [35,36]. These findings suggest that Fc-dependent functions may influence containment of Mycobacterium tuberculosis and support the development of antibody-based biomarkers, vaccines, and adjunctive immunotherapies [35,36]. Collectively, these observations support a shift from a neutralization-centric model toward a broader view in which antibody efficacy reflects the coordinated integration of Fab-mediated recognition and Fc-mediated immune orchestration [27,28,37].

### 7.2. Pathogenic Fc Functions and Antibody-Dependent Enhancement

While Fc-mediated responses often contribute to protection, they can also amplify disease under specific immunological conditions. The most prominent example is antibody-dependent enhancement (ADE), in which immune complexes facilitate uptake of pathogens by FcγR-expressing cells, potentially increasing viral replication and inflammatory pathology [38]. Dengue virus remains the best-characterized model of ADE. In this setting, sub-NAbs can promote FcγR-mediated viral entry into monocytes and macrophages, thereby increasing disease severity [38,39,40]. Importantly, ADE is not solely governed by antibody concentration but is influenced by antibody subclass distribution, Fc glycosylation, the balance between activating and inhibitory FcγRs, and local inflammatory signals, which collectively determine whether Fc engagement promotes pathogen clearance or disease enhancement [38,39]. Systems serology studies have begun to identify Fc signatures associated with protection versus enhancement in dengue and related infections. However, substantial cohort-to-cohort variability underscores the need for prospective validation before these signatures can be translated into vaccine or therapeutic design strategies [41,42,43].

SARS-CoV-2 illustrates the complexity of Fc biology in human disease. Several studies reported that highly inflammatory Fc profiles, including afucosylated IgG1 antibodies with enhanced binding to FcγRIIIa, were associated with severe COVID-19 in some patient cohorts [32]. At the same time, Fc-competent antibodies clearly contribute to viral control and disease attenuation, demonstrating that Fc-dependent activity is neither uniformly protective nor uniformly pathogenic [32,33]. Instead, clinical outcomes appear to depend on the balance between beneficial effector functions and excessive inflammatory activation. Similar patterns have been described in tuberculosis and TB-associated comorbidities, where inflammatory antibody signatures correlate with active disease and increased susceptibility among individuals with diabetes or chronic kidney disease [35]. Whether these Fc characteristics are causal drivers of pathology or biomarkers of underlying immune dysregulation remains uncertain and requires longitudinal investigation [36]. Together, these findings emphasize that successful anti-infective antibody design must not only maximize protective effector functions but also minimize Fc configurations associated with immunopathology, enhancement, or uncontrolled inflammation.

### 7.3. Systems Serology as a Platform for Rational Anti-Infective Antibody Design

One of the most important advances in contemporary antibody research has been the emergence of systems serology as a framework for defining multidimensional correlates of protection [41]. Rather than evaluating antibody titers or neutralization in isolation, systems serology integrates measurements of antigen specificity, isotype, subclass, glycosylation, FcR engagement, and effector functions using computational and machine-learning approaches to identify protective immune signatures [8]. HIV vaccine studies provide compelling proof of concept. Comparative analyses of multiple HIV vaccine regimens demonstrated that each strategy generated a distinct Fc-functional profile, and that combinations of IgG subclass distribution, FcγR binding, and Fc-dependent activity correlated with reduced infection risk, as demonstrated by the RV144 trial despite limited NAb responses [44]. These findings suggest that protective immunity may often depend on coordinated Fc-functional networks rather than on single immunological parameters and raise the possibility that reproducing favorable Fc signatures may be more achievable than inducing broadly neutralizing antibodies (bNAbs) in many infectious diseases [45].

Comparable observations have been reported for influenza, Ebola virus disease, and malaria, where protection is associated with specific combinations of antibody subclasses, glycosylation patterns, FcR engagement, and effector functions [8,27]. Current anti-infective mAbs are primarily optimized for affinity and neutralization, yet systems serology suggests that reproducing naturally protective Fc signatures may substantially improve clinical efficacy [27,41]. Rational incorporation of subclass selection, glycoengineering, and Fc optimization, therefore, represents a promising strategy for next-generation anti-infective biologics. Notably, relatively few licensed antibodies currently incorporate such disease-specific Fc design principles, highlighting a significant translational gap between mechanistic insight and clinical implementation [1,31].

## 8. Disease-Specific Fc Biology in Autoimmunity and Immune-Mediated Disorders

### 8.1. Autoantibody Subclasses as Determinants of Disease Mechanism

Emerging evidence indicates that antibody subclass is not merely a biomarker of autoimmune disease but can actively determine pathogenic potential. Experimental studies in IgG4-mediated disorders demonstrated that transferring identical antigen specificity onto different IgG subclass backbones substantially altered disease severity by modifying FcR engagement and downstream effector functions [46]. These findings establish subclass identity as a critical determinant of autoimmune pathology and reinforce the broader concept that Fc architecture can influence disease independently of antigen recognition. IgG4 is particularly illustrative of this principle. Owing to its limited complement activation, weak interaction with activating FcγRs, and unique capacity for Fab-arm exchange, IgG4 often functions as a regulatory or blocking antibody [47,48,49]. These characteristics confer protective effects in allergy, where IgG4 can antagonize IgE-mediated responses. Still, they can also drive pathology in diseases characterized by the blockade of essential signaling pathways or dysregulated immune regulation [47,48,49]. Consequently, the same properties that make IgG4 attractive for therapeutic antibodies can, under certain circumstances, contribute to the development of autoimmune disease. Clinical observations in disorders such as myasthenia gravis, membranous nephropathy, and IgG4-related disease further support the importance of subclass-specific biology and suggest that subclass profiling may provide both mechanistic insight and therapeutic guidance [47].

### 8.2. FcRn as a Therapeutic Target in Autoimmunity

Among recent advances in autoimmune therapeutics, FcRn inhibition provides one of the most direct demonstrations that manipulating Fc biology can produce meaningful clinical benefit. By preventing FcRn-mediated IgG recycling, FcRn antagonists accelerate degradation of circulating IgG and reduce concentrations of pathogenic autoantibodies [50]. Clinical trials in myasthenia gravis, immune thrombocytopenia, and related disorders have shown that FcRn inhibition can reduce total IgG levels by approximately 60–80% while producing parallel reductions in disease-associated auto-antibodies and clinically meaningful improvements in disease activity [50]. Importantly, these therapies largely spare IgM and IgA, allowing selective targeting of pathogenic IgG-mediated mechanisms [50]. Beyond their immediate therapeutic impact, FcRn inhibitors validate the concept that systemic manipulation of antibody homeostasis can be used therapeutically. However, questions remain regarding long-term safety, infection susceptibility, vaccine responsiveness, and interactions with Fc-engineered mAbs possessing enhanced FcRn affinity, such as YTE- and LS-containing variants [7,50].

### 8.3. Fc-Centric Immunomodulation Beyond Antibody Depletion

Therapeutic exploitation of Fc biology in autoimmunity extends beyond reducing pathogenic antibodies. Fc multimers, engineered Fc constructs, intravenous immunoglobulin (IVIG), and effector-silenced therapeutic antibodies represent complementary strategies for actively reshaping immune responses [51]. Engineered Fc multimers can engage inhibitory FcγRIIb and other regulatory pathways, mimicking aspects of IVIG while providing greater molecular precision and opportunities for receptor-selective immunomodulation [51]. Preclinical studies have demonstrated efficacy in models of immune thrombocytopenia, arthritis, and nephritis through mechanisms that include saturation of activating FcγRs, crosslinking of inhibitory receptors, and modulation of B-cell and dendritic-cell function [51]. Together with FcRn inhibition and effector-silenced therapeutic antibodies, these approaches illustrate the emergence of Fc biology as a therapeutic platform rather than merely a structural component of antibody molecules [7,50]. Despite this progress, comparative clinical studies evaluating different Fc-based strategies remain limited, and the field still lacks a comprehensive framework for selecting optimal Fc architectures for specific autoimmune diseases.

## 9. Disease-Specific Fc Biology in Cancer

### 9.1. Fc-FcγR Interactions as Determinants of Antitumor Efficacy

The clinical success of many therapeutic antibodies in oncology has established that Fc-mediated mechanisms are often as important as antigen recognition itself. The efficacy of several established therapeutic antibodies, including anti-CD20 and anti-HER2 agents, depends substantially on engagement of activating FcγRs expressed by natural killer cells, macrophages, and other innate immune effectors [52]. The importance of Fc engagement extends beyond direct tumor-cell targeting. Mechanistic studies in murine tumor models have shown that activating FcγRs enhances the activity of immunomodulatory antibodies targeting CTLA-4, GITR, and OX40 by facilitating the depletion or functional reprogramming of intratumoral regulatory T cells [53,54,55]. Additional investigations demonstrated that FcγR-mediated crosslinking can enhance signaling through death receptors and co-stimulatory pathways, thereby amplifying the activity of pro-apoptotic and immune-activating antibodies [56,57]. Collectively, these findings indicate that Fc engagement frequently constitutes an integral component of the therapeutic mechanism rather than a secondary contributor to efficacy [52,54].

Checkpoint blockade illustrates the context-dependent nature of Fc biology in cancer therapy. Unlike tumor-targeting antibodies, where Fc-mediated effector functions are often desirable, studies of anti-PD-1 antibodies have demonstrated that FcγR engagement can paradoxically reduce efficacy by promoting macrophage-mediated depletion of PD-1-expressing effector T cells [58]. In murine models, Fc-null anti-PD-1 variants generated more consistent T-cell activation and superior tumor control than Fc-competent counterparts across diverse immune environments [58]. These observations emphasize that optimal Fc architecture must align with the therapeutic mechanism and that Fc engagement may either enhance or undermine antitumor immunity, depending on the biological context [52,58]. Clinical data provide additional support for the therapeutic relevance of Fc biology.

Furthermore, higher-affinity variants of FcγRIIIa and FcγIIa are frequently associated with improved clinical responses or prolonged progression-free survival among patients receiving anti-CD20, anti-HER2, and other IgG1-based therapies [59]. Consistent with earlier observations regarding FcγRIIIa V158F and FcγRIIa H131R polymorphisms, these findings suggest that host immunogenetic variation can influence the clinical performance of therapeutic antibodies by modulating FcR engagement [52,59]. Although prospective validation remains limited, such observations support the emerging concept that FcR genetics may become an important component of future biomarker-driven antibody development.

### 9.2. Fc Engineering as a Platform for Therapeutic Optimization

Contemporary Fc engineering strategies seek not only to enhance effector functions but also to optimize pharmacokinetics, improve selectivity, and mitigate toxicity. A recent review highlighted how Fc engineering can modulate FcγR engagement, complement activation, FcRn interactions, and compatibility with emerging multispecific antibody architectures, noting that more than 30 Fc-engineered antibodies are either approved or undergoing clinical evaluation [60]. Similarly, three major categories of clinically relevant Fc modifications exist: glycoengineering approaches that enhance FcγRIIIa binding, amino-acid substitutions that tune FcγR and C1q interactions, and FcRn-targeted mutations that modify systemic exposure and dosing frequency [7]. Among these approaches, afucosylation is among the most successful translational examples. Removal of core fucose from the conserved Fc glycan substantially increases FcγRIIIa affinity and enhances ADCC, a strategy incorporated into antibodies such as obinutuzumab and several anti-CD38 therapeutics [60]. Experimental studies demonstrate that afucosylated antibodies can produce markedly enhanced effector activity and improved tumor-cell elimination. However, increased immune activation may also contribute to infusion-related reactions and cytokine-release events in some settings [60]. Additional engineering strategies include Fc variants containing substitutions such as S239D and I332E, which enhance activating FcγR binding, and HexaBody-like designs that promote antibody hexamerization and complement activation on tumor-cell surfaces [7,9,60]. These approaches illustrate how Fc engineering can selectively amplify specific immune mechanisms in line with therapeutic requirements.

Conversely, certain oncology applications benefit from reducing rather than enhancing Fc-mediated activity. Studies of anti-PD-1 antibodies demonstrate that limiting FcγR engagement can prevent depletion of activated effector T cells and improve therapeutic efficacy [58]. Similarly, excessive Fc activation may contribute to cytokine release, immune-related adverse events, and off-tumor toxicity [61,62]. Rational Fc engineering, therefore, increasingly aims to optimize the balance between therapeutic benefit and immune-mediated toxicity by selectively controlling the magnitude and specificity of FcγR interactions. Overall, oncology provides one of the clearest demonstrations that Fc architecture functions as a critical determinant of therapeutic outcome rather than a passive structural feature [7,52,60]. Future cancer immunotherapies are likely to incorporate increasingly sophisticated Fc design strategies that integrate Fc engineering, FcγR genetics, biomarker-driven patient stratification, and tumor microenvironment characteristics to maximize clinical benefit while minimizing toxicity [59,60].

## 10. Towards Precision Fc Pharmacology

A major emerging theme across infectious diseases, autoimmunity, and cancer is that Fc-mediated therapeutic activity cannot be adequately described by individual biomarkers alone. Systems serology studies have demonstrated that protective and pathogenic antibody responses are characterized by multidimensional Fc signatures encompassing subclass distribution, glycosylation, FcR engagement, and coordinated effector functions [8]. These observations provide a conceptual foundation for extending similar approaches to therapeutic antibody development. At the same time, studies of IgG allotypes, FcγR polymorphisms, and other host determinants indicate that therapeutic responses may be influenced by patient-specific biological factors that are largely ignored in current development paradigms [46,59]. Although available evidence does not yet support routine genotype-guided antibody prescribing, these findings suggest that future therapeutic strategies may increasingly incorporate host immunogenetic information into Fc design and clinical decision-making. Several groups have therefore proposed integrating Fc-focused biomarkers into prospective clinical trials. Potential approaches include FcγR genotyping, longitudinal profiling of antibody subclasses and glycoforms, and comprehensive assessment of Fc-dependent activities such as ADCP, ADCC, CDC, and natural killer cell activation alongside conventional pharmacokinetic and pharmacodynamic measurements [8,60]. Such strategies could facilitate identification of patient populations most likely to benefit from specific Fc architectures while improving prediction of efficacy and safety outcomes [8,17]. Collectively, these developments support the emergence of precision Fc pharmacology as a translational framework linking Fc structure, biological function, pharmacology, host genetics, and clinical outcomes. Within this framework, antibody isotypes, subclasses, allotypes, glycosylation patterns, and engineered Fc variants are no longer viewed as secondary molecular attributes but as modifiable therapeutic variables that can be deliberately tailored to disease-specific and patient-specific requirements. Realization of this vision will require systematic integration of Fc biology, translational pharmacology, biomarker development, and clinical trial design, supported by rigorous evidence from both successful and unsuccessful development programs [8,17].

## 11. Physiological Context and Emerging Technologies in Precision Fc Pharmacology

### 11.1. Age-Dependent Determinants of Fc Biology

The biological consequences of antibody Fc interactions are not static throughout life but are shaped by age-dependent changes in humoral immunity, FcR expression, and antibody glycosylation. These factors have important implications for vaccine responsiveness, susceptibility to infectious diseases, and the clinical performance of antibody therapeutics. A prospective study of primary SARS-CoV-2 infection demonstrated that both children and adults generated antibody responses capable of mediating Fc-dependent functions. However, the magnitude and kinetics of these responses differed substantially between age groups [34]. Notably, children frequently exhibited robust Fc-mediated immunity despite mild or asymptomatic infection, suggesting that age-related differences in Fc biology may contribute to divergent clinical outcomes [34]. Age-associated variation is also evident in FcRn-mediated antibody homeostasis. FcRn expression is particularly prominent during fetal and neonatal life, facilitating placental IgG transport and passive immunity, whereas endothelial and epithelial FcRn maintain IgG recycling and tissue distribution throughout adulthood [2,3,18]. At older ages, alterations in antibody glycosylation, subclass composition, and Fc-mediated effector functions have been linked to reduced vaccine efficacy and increased inflammatory susceptibility [8]. Systems serology studies further indicate that aging is associated with distinct Fc-functional signatures that may influence both infection risk and responses to biologic therapies [8]. Although current evidence remains insufficient to establish age-specific Fc engineering guidelines, these observations support including age as an important variable in future antibody development and clinical trial design.

### 11.2. Maternal–Fetal Immunity and Fc-Guided Antibody Transfer

Maternal–fetal antibody transfer represents one of the most physiologically important examples of Fc-dependent immune regulation. Placental transport of IgG is mediated primarily through FcRn and exhibits marked subclass selectivity, with IgG1 transferred most efficiently, followed by IgG4, IgG3, and IgG2 [2,3]. Emerging evidence suggests that FcRn expression levels, receptor polymorphisms, antibody subclass, and Fc glycosylation collectively influence the quantity and quality of maternal antibodies delivered to the fetus [3,18]. Recent studies of SARS-CoV-2 infection and maternal vaccination have provided compelling evidence that Fc characteristics influence neonatal immunity. Maternal IgG1 antibodies possessing specific Fc glycoforms are preferentially transferred across the placenta, resulting in cord-blood antibody repertoires enriched for functional Fc-mediated activities compared with maternal serum [32]. These findings suggest that placental transfer is not merely quantitative but may selectively enrich antibody populations with enhanced protective potential [32]. Although definitive Fc design strategies for maternal–fetal prophylaxis have not yet been established, available evidence supports the concept that optimizing subclass selection and Fc glycosylation could improve passive protection during early life.

### 11.3. Tissue Microenvironments as Determinants of Antibody Performance

The therapeutic activity of antibodies is profoundly influenced by the tissue environments in which they operate. Distinct anatomical compartments differ in receptor expression, cellular composition, barrier function, and inflammatory status, creating context-specific constraints on Fc-mediated activity. In respiratory infections, for example, antibody efficacy depends on coordinated activity across both mucosal and parenchymal compartments. Secretory IgA predominates at epithelial surfaces, whereas Fc-dependent IgG functions contribute substantially to pathogen clearance within lung tissues [28,31]. Studies of respiratory syncytial virus indicate that FcR engagement by antiviral antibodies can promote viral elimination through alveolar macrophages and natural killer cells. Still, they may also contribute to immunopathology when inflammatory responses become excessive [28,31].

The central nervous system (CNS) presents additional challenges because FcRn expression at the blood–brain barrier (BBB) regulates antibody trafficking and clearance, while interspecies differences complicate translation from preclinical models to humans [18]. Current evidence indicates that Fc engineering alone is generally insufficient to achieve therapeutically meaningful BBB penetration, and most successful neuro-immunological strategies rely on receptor-mediated transcytosis or alternative delivery approaches [18].

Within tumors, Fc-mediated activity is further shaped by hypoxia, abnormal vasculature, extracellular matrix composition, and immune-cell infiltration. Tumor-associated macrophages and natural killer cells represent major Fc-responsive effectors, yet immunosuppressive microenvironments frequently attenuate their activity [20,52]. These observations underscore the importance of evaluating Fc-optimized antibodies within disease-relevant tissue contexts rather than relying exclusively on peripheral blood-based functional assays. Collectively, these findings support the emerging concept that optimal Fc architecture is determined not only by target biology but also by the physiological compartment in which therapeutic activity is required.

## 12. Emerging Technologies Driving Next-Generation Fc Design

### 12.1. Systems Serology and High-Dimensional Fc Profiling

The development of systems serology has transformed Fc biology from a collection of individual measurements into a multidimensional framework for therapeutic discovery. By integrating antibody subclass, Fc glycosylation, FcR binding, complement engagement, and multiple effector functions using computational and machine-learning approaches, systems serology enables the identification of Fc signatures associated with protection or pathology [8,41]. Studies of HIV vaccine responses demonstrated that protective immunity can emerge despite limited neutralizing activity when antibodies possess favorable combinations of IgG subclass distribution, FcR engagement, and Fc-mediated effector functions [41,45]. Similar protective signatures have been reported for influenza, Ebola virus disease, and malaria [8]. These observations support the concept that therapeutic antibody development should move beyond optimization of antigen binding alone and instead incorporate Fc-functional profiling during candidate selection and lead optimization [7].

### 12.2. Modular Fc Engineering and Reusable Fc Platforms

A major advance in antibody engineering has been the emergence of modular Fc design strategies, in which defined Fc variants function as reusable platforms, or “chassis,” that can be combined with different antigen-binding domains [7]. Frequently employed modules include afucosylated IgG1 scaffolds, FcγRIIIa-enhancing mutations, effector-silencing variants such as LALA-based constructs, and half-life extension motifs including YTE and LS substitutions [7]. This modular approach provides a practical framework for tailoring Fc-mediated functions, pharmacokinetics, and safety profiles across diverse therapeutic indications [60]. However, Fc modules cannot be considered universally interchangeable. Interactions among Fc modifications, antigen-binding domains, bispecific architectures, and manufacturing processes can generate emergent properties affecting receptor binding, stability, aggregation, and immunogenicity [60]. Consequently, the successful implementation of modular Fc engineering requires disease-specific validation rather than reliance on a single optimal Fc template.

### 12.3. Artificial Intelligence and Computational Fc Optimization

Advances in computational biology are increasingly enabling data-driven approaches to Fc engineering. Structural modeling, molecular dynamics simulations, and in silico mutagenesis are now routinely used to predict the effects of Fc modifications on receptor binding, stability, and developability [60]. These approaches are further complemented by deep mutational scanning datasets that capture the functional consequences of thousands of Fc variants and serve as training data for machine-learning models that prioritize promising mutation combinations [60]. Although computational methods have successfully identified Fc variants with improved receptor engagement and biophysical properties, most reported applications remain at the preclinical stage, and relatively few peer-reviewed studies have linked AI-guided Fc design directly to approved therapeutics [7]. Similarly, current evidence does not yet demonstrate superiority of AI-driven engineering over conventional iterative optimization in terms of clinical outcomes [60]. Nevertheless, these technologies are dramatically expanding the accessible Fc design space and may accelerate the development of increasingly sophisticated antibody platforms.

## 13. Developability, Manufacturing, and Glycosylation as Fc-Linked Critical Quality Attributes

### 13.1. Fc Glycosylation as a Functional Determinant of Therapeutic Performance

Fc glycosylation is increasingly recognized as a central regulator of antibody activity rather than merely a manufacturing attribute. The conserved N-linked glycan within the IgG Fc region directly influences FcγR binding, complement activation, pharmacokinetics, and immunogenicity, making glycosylation one of the most consequential determinants of therapeutic antibody performance [63]. Comprehensive analyses have demonstrated that individual glycan structures exert distinct biological effects. High-mannose glycans are associated with accelerated clearance and reduced systemic persistence, whereas afucosylated glycans enhance FcγRIIIa binding and potentiate ADCC (Table 3) [63,64]. Galactosylation and sialylation further modulate complement activation and anti-inflammatory properties, illustrating how glycan composition can influence multiple dimensions of antibody function simultaneously [63,64]. Importantly, these effects are not restricted to conventional mAbs but also extend to Fc-fusion proteins, in which glycosylation of both the Fc and ligand domains contributes to receptor interactions, molecular stability, and immunogenicity [65]. These observations highlight a critical principle: Fc glycosylation should be considered an integral component of Fc design rather than a downstream manufacturing variable.

### 13.2. Bioprocess Engineering as a Tool for Fc Optimization

The growing importance of Fc glycosylation has transformed bioprocess development into an integral part of therapeutic optimization. Because glycan structures influence efficacy, pharmacokinetics, and safety, regulatory agencies classify glycosylation as a critical quality attribute requiring extensive analytical characterization and process control [66]. Advances in cell-line engineering, culture optimization, and process monitoring now enable increasingly precise manipulation of glycan profiles during manufacturing [67]. Variables including nutrient composition, carbon-source feeding strategies, culture temperature, and trace-metal concentrations can substantially alter Fc glycosylation patterns and, consequently, biological activity [67]. These capabilities have enabled the intentional engineering of Fc glycoforms rather than merely monitoring them. A TNF-α-blocking IgG1 antibody, in which timed galactose supplementation during Chinese hamster ovary (CHO) cell culture significantly increased terminal Fc galactosylation while maintaining productivity and overall product quality [68]. The resulting shift enhanced CDC without compromising other critical attributes, illustrating how relatively modest manufacturing interventions can produce biologically meaningful changes in Fc function [68]. Such studies underscore the growing convergence of process engineering and Fc pharmacology, where manufacturing decisions increasingly contribute directly to therapeutic performance.

### 13.3. Fc Quality Attributes and the Biosimilar Challenge

The importance of Fc-linked attributes is particularly evident in biosimilar development. Unlike small-molecule drugs, biosimilars must demonstrate not only structural similarity but also functional equivalence across a range of Fc-dependent activities [69]. Consequently, glycosylation profiles, FcR binding, complement activation, and pharmacokinetic behavior represent key comparability parameters during regulatory evaluation [66,70]. Fc glycosylation is one of the most closely scrutinized quality attributes, as even subtle shifts in glycan distribution can influence FcγR engagement and biological activity [66,70]. Studies evaluating adalimumab biosimilars have similarly highlighted Fc glycosylation, FcR binding, and TNF-α neutralization potency as central determinants of biosimilarity assessments [69,71,72]. Analyses comparing innovator antibodies with biosimilar products demonstrate that although most biosimilars closely replicate the reference molecule, measurable differences in glycan composition and Fc-functional characteristics can occur and require careful functional evaluation to establish clinical relevance [73]. These findings reinforce the principle that Fc-linked quality attributes are not merely analytical endpoints but mechanistic determinants of therapeutic performance.

## 14. Regulatory and Comparability Frameworks for Fc-Containing Biologics

### 14.1. Evolving Regulatory Expectations for Fc-Containing Therapeutics

The increasing structural and functional complexity of therapeutic antibodies has elevated Fc characterization from supporting analytical exercise to a central component of regulatory evaluation. Regulatory agencies now recognize that Fc-mediated properties, including FcγR engagement, complement activation, FcRn interactions, and glycosylation profiles, can substantially influence efficacy, safety, pharmacokinetics, and immunogenicity, thereby requiring rigorous assessment throughout product development. The European Medicines Agency (EMA) guideline for biosimilar mAbs establishes a stepwise, comparability-driven framework in which extensive physicochemical and functional characterization serves as the foundation for development [74]. Importantly, the guideline requires detailed evaluation of Fc-related attributes, including FcγR binding and effector functions, and emphasizes that the scope of clinical studies should reflect the degree of residual uncertainty remaining after analytical and functional comparisons [74]. Complementing this approach, broader EMA biosimilar guidance reinforces the principle that analytical similarity should serve as the primary determinant of development strategy, with non-clinical and clinical studies providing confirmatory evidence rather than independently re-establishing efficacy and safety [75]. Consistent themes are evident across international regulatory frameworks. Reviews summarizing positions from the EMA and the US Food and Drug Administration (FDA) highlight Fc glycosylation, FcγR binding, complement activation, and Fc-mediated functional activity as critical determinants of comparability assessments and clinical development requirements [76,77]. Recent regulatory analyses further emphasize the need for mechanism-based evaluation of Fc modifications [78]. Effector-enhancing and effector-silencing designs now require clear biological justification, supported by translational evidence, demonstrating that engineered Fc properties meaningfully contribute to therapeutic objectives without introducing unacceptable safety risks. Consequently, early regulatory engagement is increasingly recommended for programs incorporating novel Fc architectures, alternative isotypes, or extensive Fc engineering, enabling alignment on analytical expectations, pharmacology packages, and safety assessment strategies before late-stage development [78]. These considerations are becoming increasingly important as therapeutic platforms expand beyond conventional IgG1 antibodies to include Fc-engineered molecules, bispecific antibodies, Fc-fusion proteins, and novel isotype-based formats. Recent approvals of Fc-engineered antibodies underscore the successful translation of Fc-optimization strategies into clinical practice (Table 4) [79,80,81,82,83,84]. Collectively, these developments illustrate a broader shift in regulatory science: Fc biology is no longer viewed as a secondary molecular attribute but as a critical determinant of therapeutic performance that must be incorporated into evidence-based development strategies.

### 14.2. Analytics-First Development and Fc-Focused Risk Assessment

**Figure 3:**
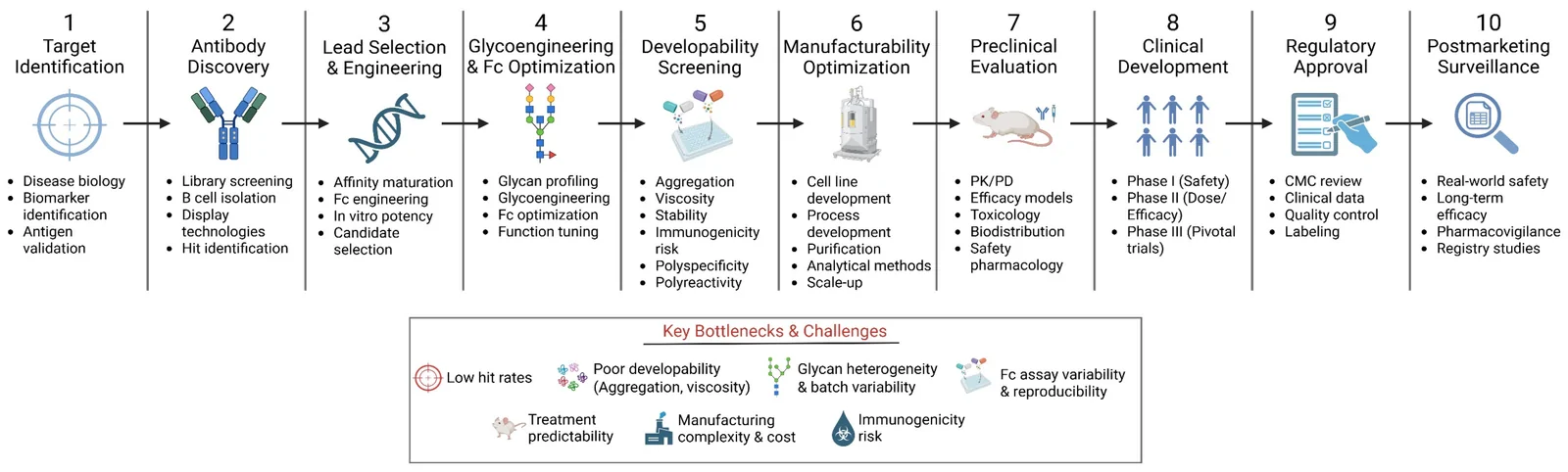
Analytics-first development pathway for Fc-optimized antibody therapeutics. The schematic outlines a ten-stage continuum from early target identification to post-marketing surveillance, emphasizing points at which Fc biology, glycosylation, and developability considerations intersect with classical drug-development milestones. The lower panel highlights recurring bottlenecks and challenges across this pathway. Created in BioRender. Joshi, D. (2026) https://BioRender.com/71x0ct2.

An important evolution in biologics regulation is the emergence of an analytics-first paradigm, in which high-resolution structural and functional characterization drives decision-making throughout development. Regulatory agencies are increasingly adopting frameworks that prioritize detailed analytical characterization, including Fc profiling, glycosylation analysis, receptor-binding assessment, and functional characterization, to identify and mitigate development risks early in the product lifecycle [85]. This approach is particularly relevant for Fc-engineered antibodies and Fc-fusion proteins, where modifications frequently influence multiple interconnected properties simultaneously. Alterations designed to enhance FcγR binding, prolong half-life through FcRn engagement, or silence effector functions may also affect tissue distribution, immunogenicity, molecular stability, and safety profiles Figure 3. Consequently, regulators increasingly expect integrated analytical datasets capable of linking structural modifications to biological outcomes [85].

Experience with Fc-engineered therapeutics further highlights the limitations of conventional safety assessment strategies. Afucosylated antibodies or FcγR-enhanced variants revealed cytokine-release syndromes or unintended depletion of non-target cell populations that were not fully predicted by standard animal studies [17]. Such observations largely reflect species-specific differences in FcγR biology and emphasize the importance of human-relevant testing systems. As a result, contemporary regulatory packages increasingly incorporate complementary approaches, including human primary-cell assays, humanized FcγR and FcRn animal models, advanced receptor-binding studies, and orthogonal biophysical analyses [17]. These methodologies are intended to improve the prediction of clinical performance while reducing uncertainty associated with highly engineered Fc constructs. Experience from biosimilar development further supports this analytical emphasis. Reviews of EMA biosimilar approvals indicate that products demonstrating high similarity in Fc-linked quality attributes and functional activity can often progress with streamlined clinical programs. In contrast, unresolved differences in glycosylation or Fc-mediated functions typically necessitate broader clinical evaluation [86]. Although no universally harmonized framework currently links specific Fc design features to predefined regulatory pathways, guidance from EMA, FDA, WHO, and other agencies increasingly converges toward a mechanistic, analytics-driven model centered on Fc biology.

## 15. Conclusions and Future Directions

While foundational studies established structural and functional differences among IgM, IgD, IgG, IgA, and IgE, as well as among the four IgG subclasses, subsequent advances have revealed how allotypic diversity, FcγR polymorphisms, FcRn biology, and glycosylation collectively shape antibody behavior in health, disease, and therapeutic intervention [2,3,24,59]. A recurring theme across infectious diseases, autoimmunity, and cancer is that Fc-mediated mechanisms frequently determine clinical outcomes as strongly as antigen recognition itself. Systems serology studies have demonstrated that protective immunity is often associated with distinct Fc-functional signatures rather than neutralization alone [8,27,28]. Autoimmune disease research has shown that FcRn-directed therapies, IgG subclass biology, and Fc-targeted immunomodulation can directly alter disease pathogenesis [46,50,51]. In oncology, FcγR engagement remains a major determinant of therapeutic efficacy. At the same time, Fc engineering has emerged as a powerful strategy to enhance or selectively attenuate immune-cell recruitment, depending on therapeutic objectives [7,52,53,60]. Despite these advances, clinical exploitation of Fc diversity remains surprisingly limited. Large-scale analyses reveal continued reliance on a relatively narrow subset of IgG1-, IgG2-, and IgG4-based architectures despite growing evidence that alternative subclasses, non-IgG isotypes, engineered allotypes, and customized Fc variants may offer advantages in specific biological contexts [19,23]. Furthermore, host genetic factors, including GM allotypes, FcγR polymorphisms, and potentially FcRn variants, remain largely absent from current drug development paradigms despite mounting evidence of their influence on therapeutic responses [3,24,59].

Unlike previous reviews that address these components independently, we propose that their collective interpretation provides the basis for a new paradigm of precision Fc pharmacology. In this framework, antibody design evolves from a predominantly target-centric process to a multidimensional, mechanism-driven strategy in which Fc-mediated biology is intentionally tailored to disease-specific and patient-specific requirements. Future therapeutic development will increasingly rely on Fc-associated biomarkers, host genetic variation, modular Fc engineering platforms, advanced glycoengineering, and systems-level immune profiling to guide therapeutic selection and optimization. Realization of this vision will require prospective clinical studies incorporating Fc-functional biomarkers, standardized analytical platforms, harmonized regulatory frameworks, and direct comparative evaluation of alternative Fc architectures within identical antigen-specific platforms. If successfully implemented, precision Fc pharmacology has the potential to transform antibody isotypes, subclasses, allotypes, and Fc engineering from supporting design variables into central determinants of rational therapeutic development across infectious diseases, autoimmunity, cancer, and next-generation biologics.

## Figures and Tables

**Table 1:** Structural and functional features of human antibody isotypes and subclasses in infectious diseases, autoimmunity, and cancer.

Antibody Isotype/Subclass	Key Structural Features	Dominant Localization	Serum Half-Life (Days)	Principal Fc Receptors and Complement Interactions	Dominant Effector Functions	Role in Infectious Diseases	Role in Autoimmune Diseases	Role in Cancer and Therapy
IgM	Pentamer linked by J chain; high hinge flexibility; high avidity antigen binding	Intravascular compartment	~5	Limited classical FcγR engagement; potent C1q binding;	Potent classical complement activation and immune complex clearance; agglutination	Rapid containment of acute viral infections such as influenza; first-line control of blood-borne pathogens	Can participate in immune complex formation and complement activation in systemic autoimmune diseases	Used as a complement activation; rare therapeutic use but informs design of highly avid multimeric antibody constructs
IgG1	Monomer; intermediate hinge length; balanced flexibility and stability	Systemic circulation and tissues	~21	High affinity for FcγRI (CD64); efficient engagement of FcγRIIa (CD32a) and FcγRIIIa (CD16a); robust C1q binding	Strong ADCC and ADCP; effective complement activation	Dominant response to protein antigens and many viral pathogens; key contributor to Fc-mediated protection in SARS-CoV-2 and influenza	Major isotype in pathogenic immune complexes in diseases such as rheumatoid arthritis and systemic lupus erythematosus	Backbone for most approved monoclonal antibodies in oncology and infectious diseases; platform for afucosylation, LS/YTE, and other Fc-engineering strategies
IgG2	Monomer with a short, rigid hinge with moderate hinge flexibility; distinct disulfide bonding; limited Fc exposure	Blood and spleen	~20	Weak interaction with most FcγRs; poor C1q binding	High-avidity binding to repetitive carbohydrate epitopes; limited cell-mediated effector function	Critical for immunity to encapsulated bacteria and polysaccharide-rich surfaces; IgG2 deficiency predisposes to such infections	Altered IgG2 responses linked to autoimmune overlap and susceptibility patterns	Occasionally used when reduced inflammatory effector function is desired; informs design of Fc-silenced formats
IgG3	Monomer with exceptionally long hinge; multiple inter-heavy-chain disulfide bonds; highly flexible	Systemic circulation	~7	High affinity for activating FcγRs; very strong C1q binding	Potent complement activation, ADCC, and ADCP; broad Fc effector breadth	Important in immunity to viral and parasitic infections; specific IgG3 allotypes confer enhanced protection in malaria and against HSV and SARS-CoV-2	Can contribute to tissue-damaging effector responses when dysregulated	IgG3-based variants of rituximab and other antibodies improve tumour cell killing and complement activation in preclinical models
IgG4	Monomer; short hinge with unstable inter-heavy-chain disulfides; capable of Fab-arm exchange with moderate hinge flexibility	Systemic circulation and sites of chronic antigen exposure (e.g., allergen-exposed tissues, tumor microenvironment)	~21	Low affinity for activating FcγRs; minimal C1q binding	Reduced ADCC, ADCP, and complement activation; anti-inflammatory immune modulation	Can accumulate in chronic viral infections, sometimes associated with viral persistence	Central in IgG4-related disease; often associated with immune tolerance rather than acute inflammation	Used for “effector-silent” therapeutic antibodies; Fc engineering (e.g., G236A, S267E) can restore ADCP and CDC while maintaining favourable safety profiles
IgA1	Monomer in serum; dimer in secretions with J chain and secretory component; high hinge flexibility; long, O-glycosylated hinge susceptible to proteases	Serum, respiratory tract, and upper gastrointestinal tract; mucosal secretions	~6	Binds FcαRI (CD89); limited complement activation; interacts with pIgR for epithelial transport	Immune exclusion at mucosal surfaces; FcαRI-dependent phagocytosis, ADCC and immune exclusion	Key in protection against respiratory and intestinal viruses such as rotavirus; long-lived IgA-secreting plasma cells support durable mucosal immunity	Aberrantly glycosylated IgA1 drives IgA nephropathy and other IgA-mediated autoimmune disorders	Emerging therapeutic format for mucosal targeting; subclass choice and glycosylation are critical for safety and efficacy
IgA2	Short, protease-resistant hinge; resistant to bacterial IgA proteases; dimeric secretory form stabilized by J chain and secretory component	Colon and distal intestinal tract	~6	Binds FcαRI (CD89); epithelial transport via pIgR; minimal complement activation	Immune exclusion, FcαRI-mediated phagocytosis, and pathogen neutralization; modulation of mucosal inflammation	Provides frontline defense against intestinal pathogens in the colon and distal gut	Differential IgA2 responses may modulate risk and phenotype in IgA-mediated diseases	Attractive candidate for engineered IgA therapeutics and mucosal vaccines in gastrointestinal infection and cancer
IgE	Monomer with four constant domains and no classical hinge; rigid Fcε structure	Bound to FcεRI on mast cells and basophils; mucosal and tissue sites	~2	High-affinity interaction with FcεRI; prolonged receptor occupancy even without antigen	Rapid degranulation, release of histamine and mediators; type I hypersensitivity responses	Critical for anti-helminth immunity; central to allergic responses	Drives allergic and atopic disease; target for biologics that block IgE-FcεRI interaction	High-potency tumor targeting with stringent safety controls
IgD	Monomer with highly flexible, protease-sensitive hinge	Co-expressed with IgM on naïve B cells; upper respiratory mucosa	~2–3	Limited Fc receptor engagement; primarily functions via B-cell receptor complex	Modulation of B-cell activation, antigen sensing, and tolerance	Contributes to the sensing of antigenic environments at mucosal entry sites	Altered IgD expression associated with dysregulated B-cell activation in some autoimmune settings	Not currently used as a therapeutic scaffold but informs understanding of B-cell biology and immune regulation

**Table 2:** Fc Receptor Binding Characteristics and Functional Consequences.

Fc Receptor	Major Cell Types Expressing the Receptor	Activity	IgG Classes Engaged	Major Functions
FcγRI (CD64)	Macrophages, DCs, Monocytes	Activating receptor	IgG1, IgG3	Phagocytosis, antigen presentation, cytokine release
FcγRIIa (CD32a)	Macrophages, DCs, Neutrophils	Activating receptor	IgG1, IgG3, IgG4	Phagocytosis, cytokine production
FcγRIIb (CD32b)	B cells, DCs, Macrophages	Inhibitory receptor	all IgG	Immune regulation, limits BCR signaling
FcγRIIIa (CD16a)	NK cells, Macrophages	Activating receptor	IgG1, IgG3	ADCC, phagocytosis
FcRn	Endothelial cells, epithelial cells, monocytes	Recycling receptor	IgG1, IgG2, IgG4	IgG recycling, transcytosis, serum half-life

**Table 3:** Fc Glycan Modifications and Biological Effects.

Glycan Modification	Structural Change	Fc Effect	Functional Consequence	Therapeutic Implications
Afucosylation	Removal of core fucose	↑ FcγRIIIa binding	↑ ADCC	Cancer therapy
Galactosylation	Addition of galactose	↑ C1q binding	↑ CDC (complement activation)	Enhanced clearance
Sialylation	Addition of sialic acid	↓ FcγRIIIa binding	Anti-inflammatory activity	Autoimmune diseases
Bisecting GlcNAc	Addition of GlcNAc at β1,4 branch	↑ FcγRIIIa binding	↑ ADCC	Stability improvement
High Mannose Content	Increased mannose residues	↑ FcγR binding/altered clearance	Reduced effector function	May reduce efficacy; immunogenicity risk

**Table 4:** Clinically Approved Fc-Engineered Antibodies.

Antibody (Brand)	Target	Engineering Strategy	Key Fc Modification	Therapeutic Area	Functional Goal
Obinutuzumab (GAZYVA^®^)	CD20	Glycoengineering	Afucosylated IgG1	Hematologic malignancies	Enhanced ADCC
Tafasitamab (MONJUVI^®^)	CD19	Glycoengineering	S239D and I332E	DLBCL	Enhanced ADCC
Nirsevimab (BEYFORTUS^®^)	RSV F protein	Half-life extension	YTE (M252Y/S254T/T256E)	RSV prevention	Extended half-life
Ravulizumab (ULTOMIRIS^®^)	C5	Half-life extension	LS (M428L/N434S)	PNH, aHUS	Extended half-life
Efgartigimod (VYVGART^®^)	FcRn	FcRn-targeting Fc fragment	M252Y/S254T/T256E/H433K/N434F	Autoimmune diseases	FcRn antagonist
Margetuximab (MARGENZA^®^)	HER2	FcγRIIIA optimization	L235V, F243L, R292P, Y300L, and P396L	Breast cancer	Improved ADCC

Abbreviations: DLBCL: Diffuse Large B-Cell Lymphoma, PNH: Paroxysmal Nocturnal Hemoglobinuria, aHUS: Atypical Hemolytic Uremic Syndrome.

## Data Availability

No new data were created or analyzed in this study. Data sharing does not apply to this article.
